# Spatiotemporal evolution of dissolved organic matter (DOM) and its response to environmental factors and human activities

**DOI:** 10.1371/journal.pone.0292705

**Published:** 2023-10-11

**Authors:** Mengyu Liu, Haihan Tian, Tao Chen, Jingyao Sun, Ruipeng Sun, Qiang Kong, Zheng Zhao, Siju Zhang, Fei Xu

**Affiliations:** 1 College of Geography and Environment, Shandong Normal University, Jinan, Shandong, PR China; 2 The Natural Resources and Planning Bureau of Weishan, Jining, PR China; 3 Shandong Provincial GEO-MINERAL Engineering Co., Ltd., Jinan, PR China; 4 Dongying Institute, Shandong Normal University, Dongying, Shandong, PR China; University of Kalyani, INDIA

## Abstract

The South-to-North Water Diversion East Project (SNWDP-E) is an effective way to realize the optimal allocation of water resources in China. The North Dasha River (NDR) is the reverse recharge section that receives water from the Yufu River to the Wohushan Reservoir transfer project line in the SNWDP. However, the dissolved organic matter (DOM) evolution mechanism of seasonal water transfer projects on tributary waters has not been fully elucidated. In this paper, the NDR is the main object, and the changes in the composition and distribution of spectral characteristics during the winter water transfer period (WT) as well as during the summer non-water transfer period (NWT) are investigated by parallel factor analysis (PARAFAC). The results showed that the water connectivity caused by water transfer reduces the environmental heterogeneity of waters in the basin, as evidenced by the ammonia nitrogen (NH_4_^+^-N) and total phosphorus (TP) in the water body were significantly lower (p<0.05, p<0.01) during the water transfer period than the non-water transfer period. In addition, the fluorescence intensity of DOM was significantly lower in the WT than the NWT (p<0.05) and was mainly composed of humic substances generated from endogenous sources with high stability. While the NWT was disturbed by anthropogenic activities leading to significant differences in DOM composition in different functional areas. Based on the redundancy analysis (RDA) and multiple regression analysis, it was found that the evolution of the protein-like components is dominated by chemical oxygen demand (COD) and NH_4_^+^-N factors during the WT. While the NWT is mainly dominated by total nitrogen (TN) and TP factors for the evolution of the humic-like components. This study helps to elucidate the impact of water transfer projects on the trunk basin and contribute to the regulation and management of inter-basin water transfer projects.

## 1 Introduction

The South-to-North Water Diversion Project (SNWDP) was one of the major projects of inter-basin water transfer in China [1]. The eastern route of SNWDP (SNWDP-E) was a major measure to effectively alleviate the shortage of water resources in the Beijing-Tianjin-Hebei region and the Shandong Peninsula [2]. Shandong was the only river-sea intersection area in the Yellow River Basin (YRB) and an important area for ecological protection and restoration in the lower reaches of the Yellow River [3]. The pollution control of the SNWDP-E was the core, and the effect of pollution control was directly related to the success or failure of the SNWDP. The water transfer project from Yufu River to Wohushan Reservoir is the Jinan section of the SNWDP-E, which has a reverse recharge effect on the North Dasha River (NDR) to ensure its hydrological ecology during the dry season. The NDR was located in Jinan City, Shandong Province. It belonged to the right bank tributary of the lower Yellow River which was 54.3 km long and crosses the city.

Through the inspection of the cross-sections of rivers exceeding the standard in China, it was found that there are different degrees of organic matter exceeding the standard in the river systems of the whole country [4]. The NDR was similar to the Haihe River Basin and the middle and lower reaches of the Yellow River [5,6]. It was greatly disturbed by human activities. Under the background of the gradual strengthening of human activities, the shortage of water resources and the intensification of the contradiction between supply and demand, coupled with environmental problems such as eutrophication and large-scale cyanobacterial outbreaks in recent years [7]. As a tributary of the Yellow River and a recharge tributary of the Jinan section of the SNWDP-E, the study of its pollution status is of great significance to the study of the nature of pollution in the larger Yellow River basin (YRB). Meanwhile, there are few studies on the impact of water transfer projects on tributaries along the project. It was necessary to improve the pollution-carrying capacity of the NDR, diagnose the ecological status of the YRB in Shandong Province, and comprehensive ecological restoration of the basin environment.

At present, the research on organic matter in water mostly focuses on conventional indicators such as chemical oxygen demand (COD) and biochemical oxygen demand (BOD_5_) [8], which could only comprehensively reflect the total amount of organic matter in water, and was difficult to further understand the composition of organic matter. Besides the reagents used were easy to cause secondary pollution. Fluorescence spectroscopy developed in recent years can distinguish the composition and source of organic matter in water [9,10]. Dissolved organic matter (DOM) exists in various natural water bodies and has an important impact on terrestrial and aquatic ecosystems [11]. In urban polluted river water, DOM accounts for 30% ~ 40% of the total organic matter, mainly humic acid, amino acid, hydrophilic organic acid, carbohydrate, and organic pollutants produced by human activities [12]. In recent years, DOM has attracted wide attention due to its toxicity and ecological risk. Yuan et al. [13] found that DOM has complexation characteristics with Sulfadiazine. Li et al. [14] found that DOM can participate in the formation of mutagenic disinfection by-products. The source of DOM also affects the migration and transformation of harmful organic pollutants [15], such as inhibiting the oxidation of methylbenzene [16]. Chen et al. [17] found that different sources and environmental factors of DOM can lead to trade-offs in the conversion efficiency of micropollutants in water by studying the removal and degradation process of DOM in micropollutants. Three-dimensional fluorescence spectroscopy (3DEEM) is considered to be the simplest and most effective method to study the composition and source of DOM because of its simplicity, sensitivity, and low cost [18]. The fluorescence regional integration (FRI) method is mostly used to analyze DOM components by existing studies [19,20]. Fu et al. [21] characterized DOM components by FRI and found that the fluorescence regional integration (FRI) method is more suitable for indicating the content of high water-soluble organic matter in industrial and domestic wastewater. The parallel factor analysis (PARAFAC) can decompose the 3DEEM of complex mixtures into their respective fluorescent components by using appropriate model levels to identify the number and type of fluorophores and their abundance information [22], to understand and control the process of DOM concentration and its characteristics. The PARAFAC has less research on large-scale watersheds and cross-basins. However, most of these studies only sampled and analyzed a certain period or a section of the river. The spatiotemporal optical properties and source analysis of the DOM of the mainstream are not fully understood.

The NDR is a typical seasonal river with poor oxygen-enrichment capacity in winter, while its impact on the mainstem from seasonal water transfer as a tributary of the Yellow River in the SNWDP-E is unclear. In summary, this study takes the NDR as the research object, with the objectives of (1) studying the differences in the composition and distribution of spectral characteristics of the mainline tributaries before and after the water transfer period of the SNWDP-E; (2) investigating the mechanism of environmental factors on DOM components and the impact of water transfer projects on the study area. This study provides a new perspective for studying the ecological effects of water transfer and diversion projects and has important implications for future ecological management.

## 2 Materials and methods

### 2.1 Study area and water sampling

Jinan City (Shandong Province, China) is located in the south by Mount Tai and in the north by the Yellow River. The rivers belong to three major water systems, namely the Yellow River, the Huai River, and the Hai River, and are located in the lower reaches of the Yellow River, which is connected to the project of leading water from the Yellow River to Qingdao and guarantees more than 80% of the city’s water for production and living. The rivers in this study area (NDR) were specially selected to represent the possible point source pollution areas, i.e. reservoir area, residential area, and excursion area, with two sampling points in the reservoir area, two sampling points in the residential area, and three sampling points in excursion area, totaling seven sampling points. The geographical location of the sampling points is shown in Table 1.

**Table 1:** Geographic coordinates of sampling points for each functional area (reservoir areas (1, 2), residential areas (3, 4), and excursion areas (5, 6, 7)).

sampling points	geographical location	
1	36°29’24.57" N	116°50’53.54" E
2	36°29’58.14" N	116°49’59.22" E
3	36°30’37.42" N	116°49’03.82" E
4	36°31’19.97" N	116°48’43.03" E
5	36°31’40.91" N	116°48’25.79" E
6	36°32’06.29" N	116°48’12.29" E
7	36°32’39.45" N	116°48’03.32" E

The water samples were collected twice during the winter water transfer period, December 2021) and the summer flood period (end of water transfer, June 2022), with three parallel samples collected at each sampling point, and the standard deviation values were calculated (Note: Time was coded as water transfer period (WT) from upstream to downstream as W1-W7, and non-water transfer period (NWT) was coded as N1-N7.). Sample collection was carried out in strict accordance with the HJ494-2009 water quality sampling technical guidance, while the environmental data such as temperature, pH, dissolved oxygen (DO), and oxidation-reduction potential (ORP) were measured during the water sample collection process, and the surrounding environment was recorded. The collected samples were placed in a 4°C environment for low-temperature storage and returned to the laboratory for analysis within 24 h.

### 2.2 Water quality measurement

The collected water samples were filtered by the 0.45 μm filter membrane and measured immediately. COD, ammonia nitrogen (NH_4_^+^-N), total nitrogen (TN), and total phosphorus (TP) were determined following the study of Xu et al. [23]. Specific measurements were shown in S1 Table.

The single-factor index (Pi) method is the most simple and intuitive water quality evaluation method commonly used at present. The comprehensive water quality identification index (P_T_) method can not only evaluate the water quality category but also make the quantitative comparison [24]. The water quality indicators were analyzed by using the actual detection value (Si) and the standard threshold (Ci) of water quality class III of the water transfer project. The calculation methods in this study are as follows:

$$P_{i}=\frac{C_{i}}{S_{i}}$$ (1)

$$P_{T}=\frac{1}{n}\overset{n}{\underset{i=1}{\sum }}P_{i}$$ (2)

where n is the number of water quality indicators.

### 2.3 UV-Vis absorption and fluorescence spectroscopy analyses

The DOM was measured by a fluorescence spectrophotometer (F-7000, Hitachi, Japan) at excitation wavelengths of 200–400 nm, emission wavelengths of 200–500 nm, and step sizes of 2 nm. Before each measurement, ultrapure water was used as the experimental blank, and Raman normalization was performed to remove instrumental errors caused by the measurement of the 3DEEM at different times, after which the fluorescence spectra were obtained by correcting the region of the 3DEEM affected by scattering by interpolation. Specific parameter settings refer to Xu et al. [25].

The number of fluorescent components at each sampling point was determined using PARAFAC analysis, and the model was validated by half-analysis and residual validation. The spectral indices calculated in this paper include fluorescence index (FI), biological index (BIX), and humification index (HIX). The calculation method refers to the research of Liu et al. [26]. BIX and HIX are commonly used to characterize the source of DOM in water bodies. BIX characterizes the ratio of organic matter of microbial origin to organic matter of exogenous origin, and the larger the BIX value, the higher the proportion of recent autogenous fraction [27]. The HIX index is based on the principle that as the degree of humification increases, it leads to a decrease in the H: C ratio and thus a red shift in the emission spectra of fluorescent molecules [28]. The higher HIX index indicates a higher degree of humification and stronger aromaticity of DOM.

### 2.4 Statistical analyses

The DOMflour v1.7 toolkit developed by Stedmon and Bro [29] was performed using MATLAB software for PARAFAC and FRI. The location map of sampling points was performed using ArcGIS software. The correlation analysis, principal component analysis (PCA), and redundancy analysis (RDA) were performed using the Origin toolkit. We performed a linear transformation of the data used in the RDA analysis to better express the relationships between the variables. In this case, the environmental factors were used as explanatory variables, the DOM index as response variable, and the distribution of sampling points as monitoring indicators. Multiple linear regression analysis and the analysis of variance (ANOVA) were performed using SPSS software. Origin 2021 software was used to make related graphics.

## 3 Results

### 3.1 Presentation of water quality parameters

The field test data showed that the pH is between 8.07 and 8.38 in the water transfer period (WT), and between 8.38 and 8.65 in the non-water transfer period (NWT), which is weakly alkaline in general, and the pH is higher in NWT. The DO content all meets the standard of surface water category III, and the average value of DO is 11.75 mg/L in WT, while it is 9.01 mg/L in NWT.

**Fig 1:**
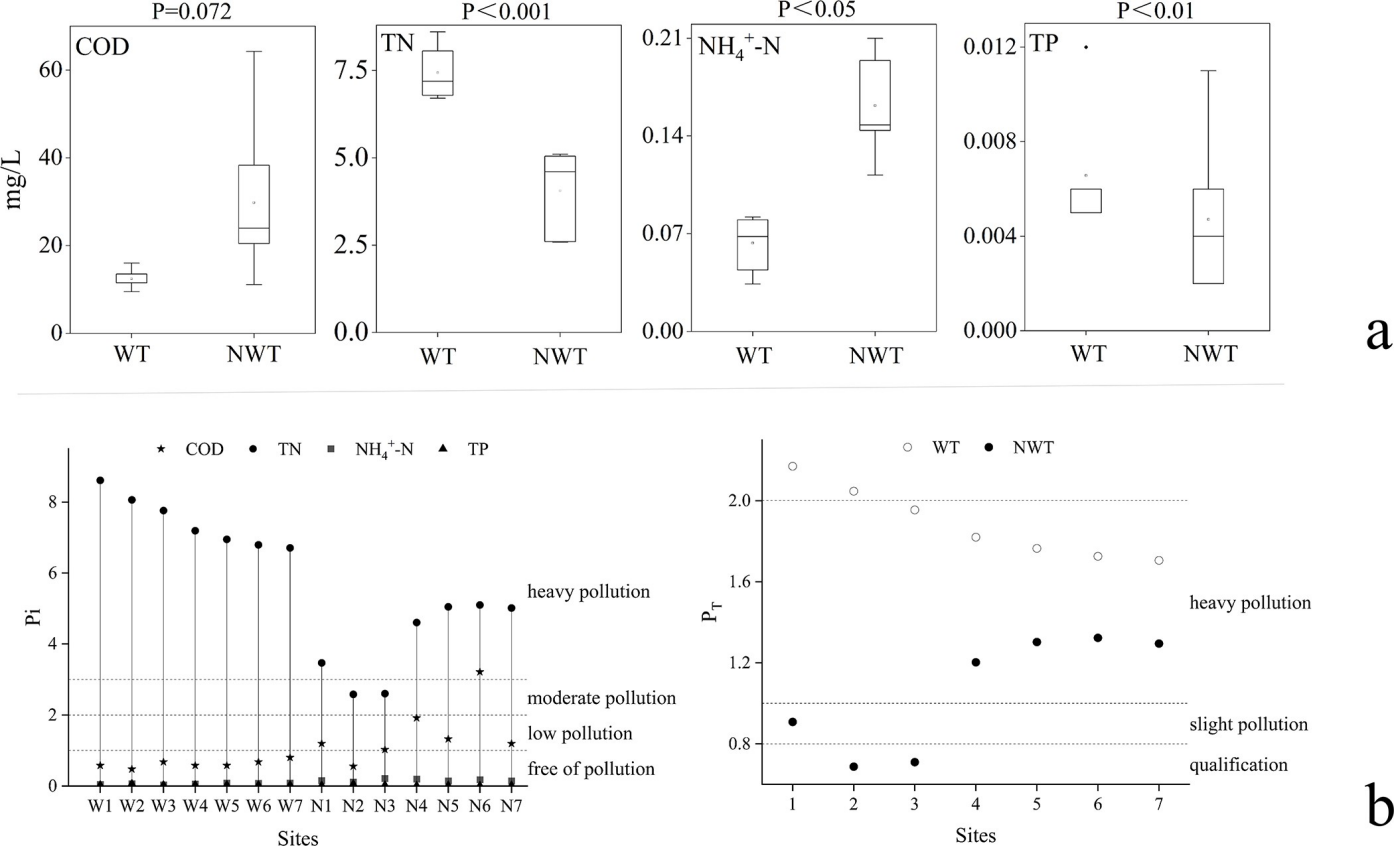
WT and NWT water quality characteristics (a) and evaluation (b) of NDR. (Pi: The single factor index; P_T_: The comprehensive index; codes are period (W/N) + site number (1–7)).

The water quality of the NDR during the WT, and the NWT analysis is shown in Fig 1. The TN concentration had highly significant differences in different periods (p<0.001), and the exceedance times were lower in NWT than in WT; and COD had no difference (P = 0.072). The NH_4_^+^-N has a general significant difference (p<0.05), and the NWT is slightly higher than the WT; The TP has a significant difference (p<0.01) in the concentration of different periods.

### 3.2 Characteristics of DOM fluorescence components

**Fig 2:**
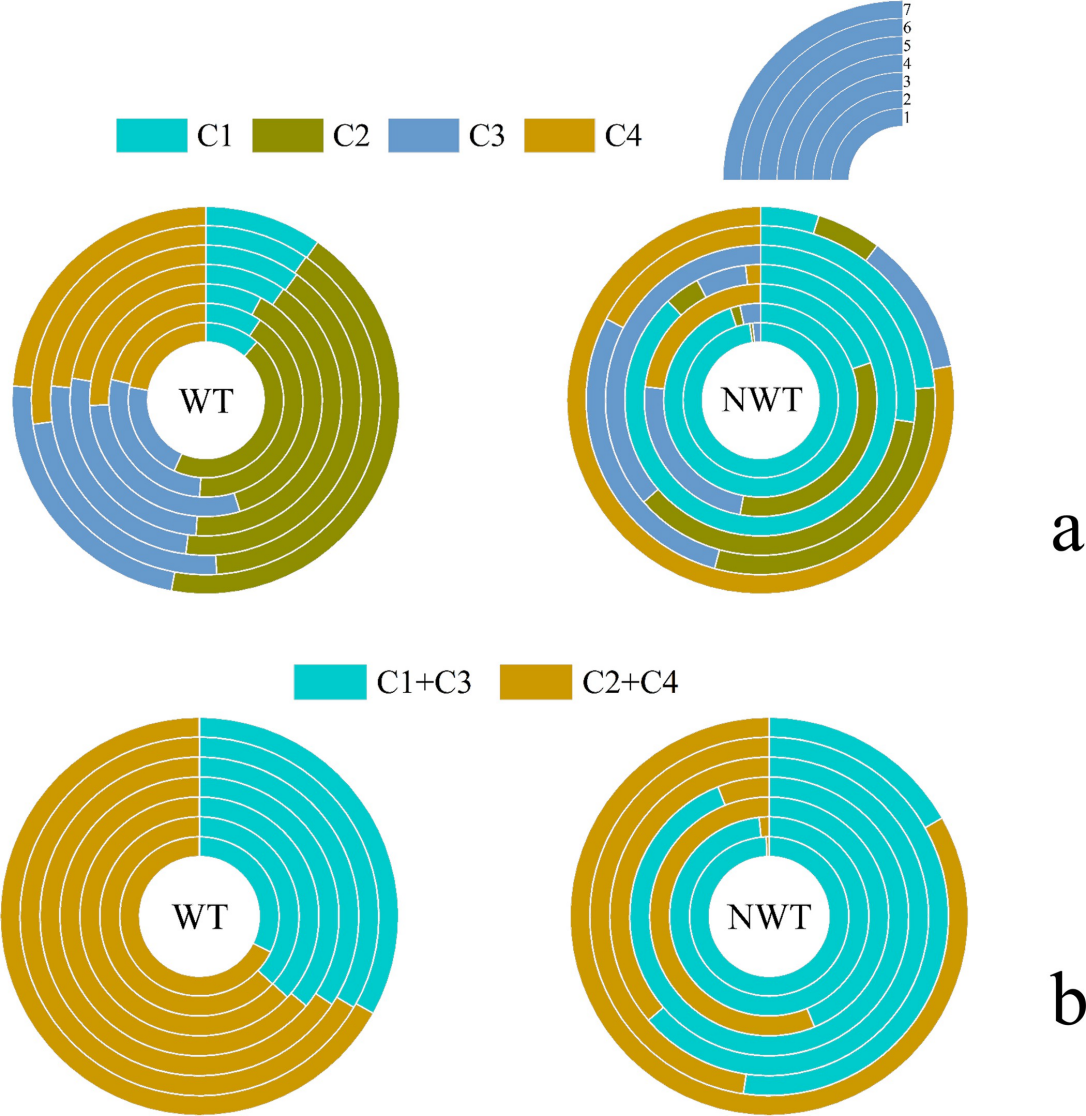
Percentage of fluorescent components at each sampling point (a is the proportion of each component, b is the proportion of easily degradable protein-like components (C1+C3) and humic-like substances (C2+C4)).

**Fig 3:**
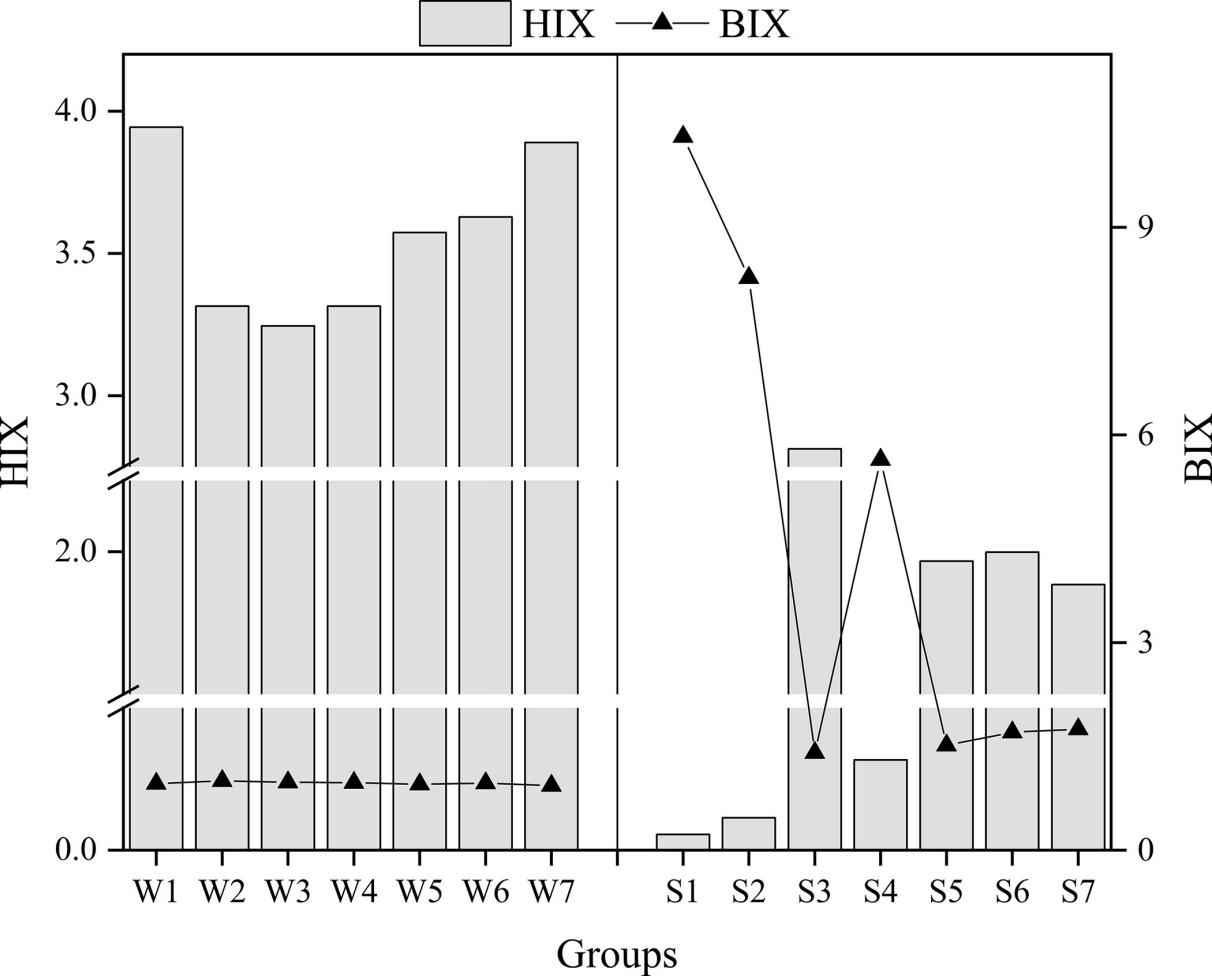
BIX, HIX index of each sampling point.

The spectral characteristics of the four components of DOM in the NDR analyzed by PARAFAC are shown in S1 Fig. The maximum fluorescence intensity (Fmax) of each component was calculated from the model analysis results and the relative content of each fluorescent component is shown in Fig 2. BIX and HIX are commonly used to characterize the source of DOM in water bodies, and the results of this study are shown in Fig 3.

The four components were compared with the Openfluor with a minimum confidence level of 0.95. The C1 component was obtained as a tryptophan-like component in the protein-like component, the C2 component was a terrestrial humic component, the C3 component was a tyrosine-like component, and the C4 component was a microbial humic component.

The composition of DOM was mainly dominated by C2, with C3 and C4 evenly distributed during the WT, and C1 during the NWT. The content of protein-like components (C1+C3) showed that the WT < NWT, while the content of humic-like components (C2+C4) showed that the WT > NWT.

The BIX values in the WT ranged from 0.935 to 1.001 with a mean value of 0.968, while the BIX values in the NWT ranged from 1.517 to 10.31 with a mean value of 4.371. The BIX index showed the reservoir area > excursion area > residential area in different functional areas. The HIX values of the sampling points in the WT ranged from 3.245 to 3.944, with a mean value of 3.559, while the HIX values in the NWT ranged from 0.055 to 2.813, with a mean value of 1.307. The HIX index showed the excursion area > residential area > reservoir area in different functional areas.

### 3.3 Correlation analysis on DOM index

**Fig 4:**
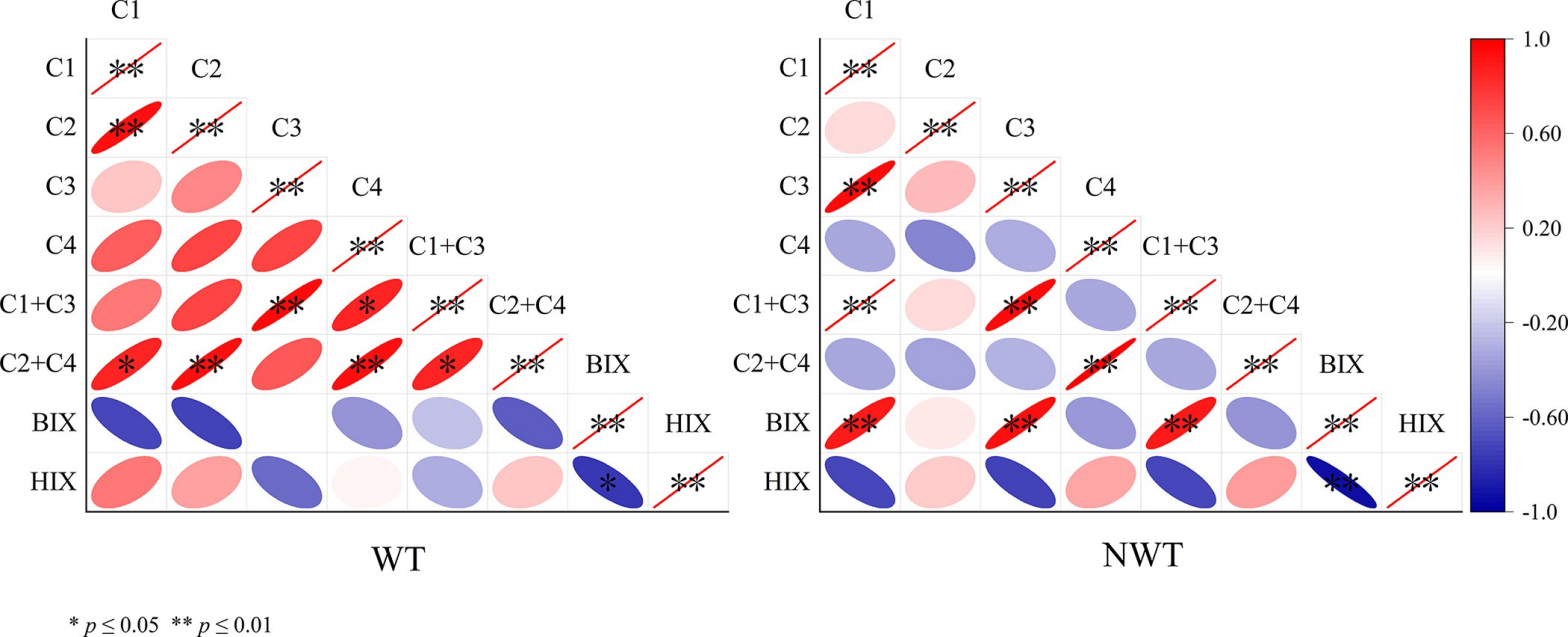
Correlation analysis between DOM and spectral index. (*, p≤0.05, significant difference; **, p≤0.01, highly significant difference. The more linear the ellipse, the closer the correlation index is to one).

The correlation between the DOM fluorescence parameters was determined by using the fluorescence components as one set of the data matrix and the fluorescence indices as another set of data matrix for correlation analysis (Fig 4), and the strength of the correlation was determined by combining the correlation matrix data.

The positive correlation between the C1 and C2 components during the WT was very significant, while the C1 and C3 components had a strong positive correlation during the NWT. The endogenous input of C1 and C3 components was significantly negatively correlated with the microbially produced C4 component during the NWT.

Between the components and the spectral indices, all components were negatively correlated with the BIX index during the WT, while all were negatively correlated with the BIX during the NWT except for the C3 component. C1 and C3 were negatively correlated with the HIX index during the NWT, C2 was positively correlated with C4, while all were positively correlated during the WT except for the C3 component.

### 3.4 RDA analysis of DOM index with environmental factors

**Fig 5:**
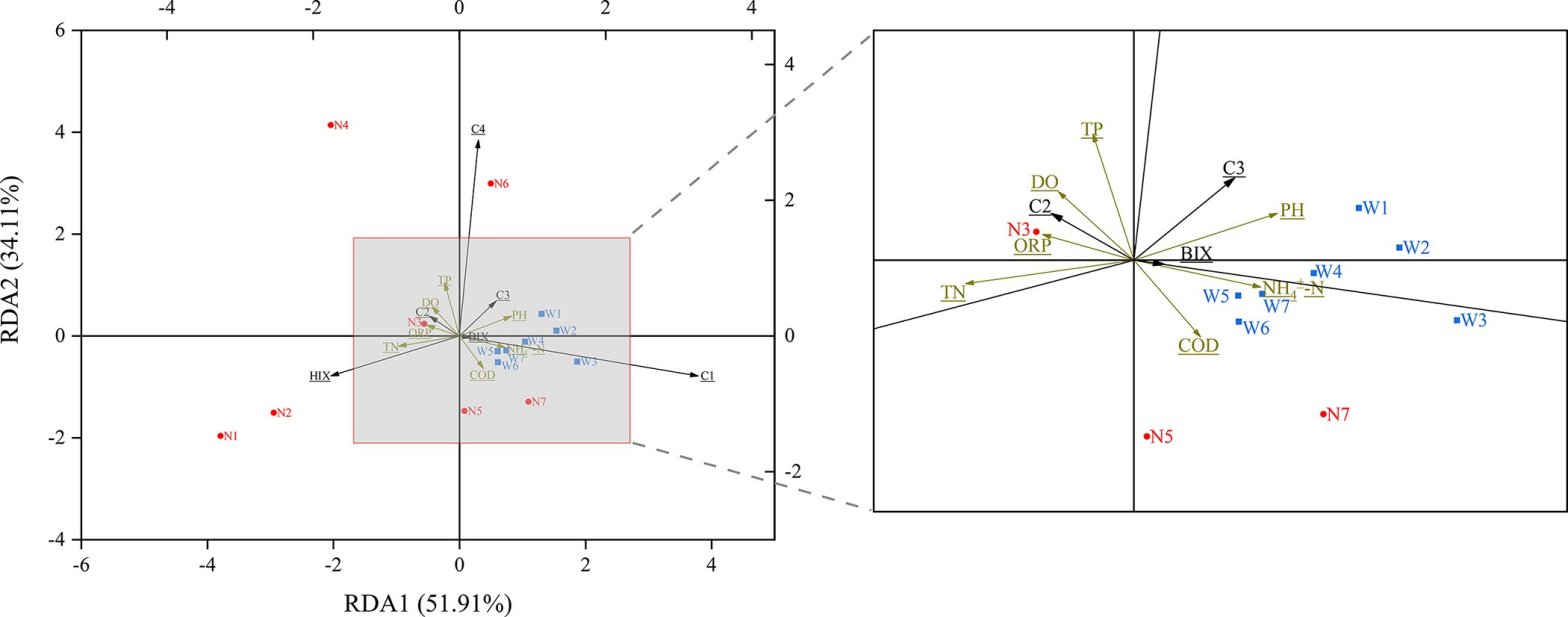
RDA analysis of DOM and environmental factors. (The breen for explanatory variables, black for response variables, blue squares for WT sampling points, and red dots for NWT sampling points).

The results of the RDA are shown in Fig 5. The correlation coefficients are shown in S2 Fig. The results of the multiple regression analysis are shown in Table 2.

**Table 2:** Regression analysis of DOM and environmental factors.

		COD	TN	TP	NH_4_^+^-N	R^2^
WT	C1	-0.155	0.021	-0.801**	1.251**	0.988**
	C2	-0.086	-0.517	-0.439	0.680	0.952*
	C3	-0.414	-1.777*	0.642*	-1.214*	0.948*
	C4	-0.037	-1.177	-0.003	-0.413	0.638
	C1+C3	-0.424	-1.665*	0.362	0.831*	0.938*
	C2+C4	-0.059	-0.889	-0.249	0.162	0.872
	BIX	-0.602	-0.489	0.692	-1.045	0.603
	HIX	0.921*	1.570**	-0.727*	1.736**	0.973*
NWT	C1	-0.568	-0.952	-1.253	-0.266	-0.102
	C2	1.003	-1.397	-0.010	-0.220	-0.014
	C3	-0.578	-0.498	-0.415	-0.124	-0.344
	C4	0.826	0.189	1.487	-0.445*	0.884
	C1+C3	-1.141	-0.562	-1.288	-0.079	-0.197
	C2+C4	-1.086	0.817	0.401	1.192	0.883
	BIX	-0.720	-0.589	-1.405	-0.611	-0.151
	HIX	-1.300	1.143	0.047	1.115	-0.808

Note

*, p≤0.05

**, p≤0.01.

As shown in Fig 5, RDA1 and RDA2 together explain 86% of the variability. The difference in the distribution of each sampling point between the two periods is obvious. The distribution of each sampling point during the WT was concentrated, mainly showing the evolution of C1 and C3 components dominated by COD and NH_4_^+^-N. The distribution of sampling points during the NWT differed significantly by functional regions, with S1 and S2 in the reservoir area being significantly correlated with the HIX indexes, and S3 and S4 in the residential area mainly being dominated by TN, TP, and DO in the evolution of the C2, whereas S5, S6, and S7 located downstream, representing the excursion area.

Multiple regression analysis of DOM components and environmental factors (Table 2) revealed that C1 and C2 were significantly and positively correlated with NH_4_^+^-N with correlation coefficients of 1.193 and 0.81 during the WT, respectively. C3 and C4 were significantly and negatively correlated with TN with correlation coefficients of -1.718 and -0.814, respectively. The C1 and C3 were significantly negatively correlated with NH_4_^+^-N, TN, and TP, while C2 was significantly negatively correlated with TN and positively correlated with NH_4_^+^-N during the NWT. The C4 was positively correlated with TN and significantly negatively correlated with COD during the NWT, while the opposite was true during the WT.

## 4 Discussion

### 4.1 Analysis of basic water quality parameters

The determination of water quality parameters plays an important role in determining the pollution level of water bodies and the status of pollutants contained in the water bodies [30]. The results of the single factor index (P_i_) and comprehensive index (P_T_) identification evaluation method as shown in Fig 1B.

Combined with the P_i_ index and P_T_ index evaluation for analysis, the results found that the overall water quality during the NWT is slightly better than the WT, while the WT presents a more stable water quality state. And by the P_i_ index found that TN is the key factor affecting the P_T_ index. Due to the contradictory nature and different hazards of TN and NH_4_^+^-N levels and standards in water, some studies have pointed out that TN is no longer used as a pollution indicator but is considered as a monitoring indicator [31], and the results of the P_T_ index evaluation by removing the TN factor are shown in the S3 Fig. The results found that the water quality during the WT is significantly better than the NWT, and the WT shows a clear relationship with the transfer trunk canal, and the water quality from the transfer entrance to the upper reaches of the river gradually improves, indicating the improvement effect of SNWDP on water quality and ecology in the receiving area. In addition, although the NDR is a seasonal river, the water quality during the WT is gradually improving, which also indicates the optimization of water allocation and ecological replenishment in the receiving area by the SNWDP.

### 4.2 Spatial and temporal distribution characteristics of DOM

#### 4.2.1 Sources of DOM

Amaral et al. [32] found that microbial activity influences the evolution of DOM through the study of DOM in shallow lakes, and the C1 (tryptophan-like component) corresponding to the present study are mainly derived from plankton and are related to indigenous production. Lin and Guo [33] found that the humic-like components were predominantly found in the river by studying the DOM of the river, while the C2 (humic-like component) corresponding to this study was mainly derived from terrestrial inputs. Catalá et al. [34] found that the tyrosine-like group was easily converted by consumption through the study of DOM in the ocean, and the C3 (tyrosine-like component) corresponding to the present study was mainly derived from algal degradation. The C4 in the study by Yamashita et al. [35] corresponding to the present study was mainly derived from microbial degradation of humic-like substances.

There was a significant difference between WT and NWT in the BIX index (Fig 3), and the difference was more significant between upstream and downstream in the NWT. The BIX values in the NWT were significantly higher than those in the WT, showing strong authigenic characteristics, mainly generated by biological and bacterial activities, which also corresponded to the increase in microbial activity in summer leading to a significant increase in C1 component at each sampling point. During the WT, the BIX values ranged from 0.935 to 1.001 with a mean value of 0.968, and showed more stability from upstream to downstream indicators. It was indicated that the stability of the index representation was achieved due to the dual role of recharge and optimization of the water transfer project in the waters under study.

The WT is subject to the relatively small input of exogenous runoff, and the good water quality of the SNWDP. The HIX values in the NWT were significantly lower than those in the WT, showing weak humic characteristics, DOM was mainly of biological or aquatic bacterial origin, and endogenous input due to planktonic degradation was the main source of DOM in the water column at the sampling points. The biological activity of the water column was also higher, and the results also corresponded to a significantly lower C2 and C4 humic component in the NWT than in WT.

#### 4.2.2 Distribution of DOM

It was found by the fluorescence spectrogram of each sampling point (S4 and S5 Figs) that the fluorescence intensity in the NWT was significantly higher than that in the WT (P < 0.05). By comparing the relative contents of each fluorescence component (Fig 2A), it can be seen that the C2 component dominates at each sampling point during the WT, and the C3 and C4 components are evenly distributed. It indicates that the DOM of water bodies during the WT mostly comes from terrestrial sources, which is discharged into the water bodies through the process of precipitation infiltration and seepage in the soil. In addition, the sediment and phytoplankton carried by the water transfer process constitute the source of water body DOM during the WT [36]. The proportion of the C2 component showed a pattern of upstream < downstream, which is consistent with the results of the evaluation of the water quality P_T_ index (S3 Fig). The sampling point in the NWT is dominated by the C1 component, which is most obvious at the source of the river. The C1 component is mostly derived from native and planktonic organisms, and the sampling points also observed that there are many fishing areas at the source, which is one of the main sources of DOM from this sampling point by artificial fish feeding and fish activities. In addition, the conversion from humic acid to fulvic acid due to strong microbial activity in summer is one of the reasons why the fluorescence intensity of fulvic acid is significantly higher in the NWT than in the WT. Besides, the increased water flow during the NWT makes the downstream more susceptible to the influence of pollutants from the upstream. The downstream C2 component is higher than the upstream, we found that the downstream is mostly the excursion area, and the NWT is rainy, which is the main reason for the increase of this component.

As shown in Fig 2B, the percentage of easily degradable protein-like substances (C1+C3) showed significant differences in the period of no use, and the percentage was higher in the NWT than in the WT. The percentage of humic-like substances (C2+C4) was higher in the WT than in the NWT. The high temperature in summer and the strong metabolism of microorganisms and algae may cause the proportion of protein-like substances to increase. And the increase of bioavailable protein-like substances in the environment also stimulates the metabolism of microorganisms [37]. For the upstream and downstream components in this study area, the percentage of (C1+C3) increased gradually from upstream to downstream during the WT, while the NWT showed differences between different functional areas. The proportion of (C2+C4) in the excursion area is the highest. This also indicates the ecological role of spring replenishment and source preservation during the WT of the SNWDP and the stabilizing effect on the water bodies of the trunk line.

### 4.3 Evolutionary characterization of DOM

#### 4.3.1 Conversion of DOM components

Among the components, the two periods showed differential patterns. The positive correlation between C1 and C2 components during the WT was very significant, indicating that the source of organic pollution during the WT was mainly riverine input carried by the transfer project. C1 and C3 components had a strong positive correlation during the NWT, indicating that the summer algal outbreak had a great impact on the organic pollution of the water body, and C1 and C3 components had similar sources or contained factors with a similar degree of influence on their concentrations. The endogenous input of C1 and C3 components was significantly negatively correlated with the microbially produced C4 component during the NWT, and the decomposition of algae led to a decrease in the DO content of the system, which to some extent weakened the microbial action [38].

Microbial activity in summer causes organic substances such as protein substances and lipids to form humic acid-like substances through aromatization and condensation, and later to humic acid to become fulvic acid [39]. Thus all components were negatively correlated with the BIX except for the C3 component in the NWT. The C1 and C3 were negatively correlated with the HIX index in the NWT, C2 was positively correlated with C4, while all were positively correlated in the WT.

#### 4.3.2 Influence of environmental factors on the conversion of DOM

RDA analysis by qualifying environmental factors (Fig 5) found that the distribution of the sampling sites during the NWT was characterized by a significant functional division. However, the overall water quality in the WT is more stable.

Relationships between explanatory and response variables: C1 was positively correlated with NH_4_^+^-N and COD, C3 was positively correlated with NH_4_^+^-N and TP, C2 was significantly positively correlated with TN, TP, and DO, and C4 was positively correlated with TP and DO. The pH was significantly correlated with C2, C3, and C4. The BIX was positively correlated with C1, C3, and NH_4_^+^-N, and COD, while the HIX was positively correlated with C2 and TN, DO had a significant correlation.

Relationships between sampling points and variables: The distribution of each sampling point was concentrated, which was mainly characterized by the evolution of C1 and C3 components dominated by COD and NH_4_^+^-N during the WT. Meanwhile, the BIX index could indirectly indicate the distribution pattern of each sampling site.

The distribution of sampling points during the NWT differs significantly from each functional area. The S1 and S2 in the reservoir area are related to the HIX indicators, S3 and S4 in the residential area are mainly dominated by TN, TP and DO in the evolution of C2 components, while S5, S6, and S7 in the downstream area representing the excursion area are similar to the distribution area of the sampling points during the water transfer period, which indicates that the water quality pattern of water quality located in the reversed recharge point during the transfer period presents a common point with that of the waters during the water transfer period. It indicates that the DOM is closely related to the nitrogen cycle [40]. Indicating that the HIX, TN, TP, and DO for C2 and C4 were the main influencing factors in the NWT, and mainly BIX, COD, NH_4_^+^-N, and pH for C1 and C3 in the WT.

Multiple regression analysis of DOM components and environmental factors (Table 2) revealed that C1 was positively correlated with NH_4_^+^-N and negatively correlated with TP with correlation coefficients of 1.251 and -0.801 during the WT, respectively. The C3 was significantly and negatively correlated with TN and NH_4_^+^-N with correlation coefficients of -1.777 and -1.214, while positively correlated with TP with correlation coefficients of 0.642, respectively. Easily degradable substances (C1+C3) were significantly negatively correlated with TN, while positively correlated with NH_4_^+^-N with correlation coefficients of -1.665 and 0.831.

However, the distribution of sampling points during the NWT showed dispersion in different functional areas, making the results of the multiple linear regression analysis present insignificant results. Combined with the RDA analysis, it can be obtained that the C4 was significantly negatively correlated with NH_4_^+^-N with correlation coefficients of -0.445. The seasonal rhythm of microbial action was indicated, with strong microbial action in summer and DO provide by algae, making aerobic nitrification strong and anaerobic denitrification weak [41].

In summary, it is shown that the composition of DOM can be predicted from the water quality condition of rivers, DOM can drive the nitrogen and phosphorus elemental cycle of water bodies [42], and the correlation and regression analysis between DOM and water quality can explore the relationship between DOM and water quality on the one hand, and also indicate the evolution of DOM. This could be with the help of correlation for monitoring and river environment protection.

## 5 Conclusion

This study reveals the importance of the South-North Water Transfer Project to the mainline tributaries by studying the differences in water quality parameters, DOM distribution, and the contribution of environmental factors. The results of the study show that there are significant differences in DOM composition and distribution with changes in hydrological conditions. The fluorescence intensity of DOM was significantly lower in the water transfer period than in the non-water transfer period and was mainly composed of humic substances generated from endogenous sources with high stability. While the non-transfer period was disturbed by anthropogenic activities leading to significant differences in DOM composition in different functional areas upstream and downstream. Indicating that the hydrological connectivity of interconnected habitats caused by water transfer reduces the environmental heterogeneity of waters in the basin. DOM and water quality correlations reveal the importance of environmental factors on the evolution of DOM. The evolution of the protein-like components is dominated by COD and NH_4_^+^-N factors during the transfer period. While the non-transfer period is mainly dominated by TN and TP factors for the evolution of the humic-like components. This study deepens the equivalence analysis of pollution indicators and biological indicators, which is important for the regulation of water transfer projects in the basin.

## Supporting information

S1 Fig PARAFAC analysis of fluorescence spectra of components of NDR. (TIF)

S2 Fig Correlation coefficients of DOM and environmental factors. (TIF)

S3 Fig The comprehensive index evaluation by removing the TN factor. (TIF)

S4 Fig FRI analysis of fluorescence spectra at each sampling site during the water transfer period (WT). (TIF)

S5 Fig FRI analysis of fluorescence spectra at each sampling site during the non-water transfer period (NWT). (TIF)

S1 Table Water quality parameters and reference methods used. (DOCX)
