# Supplementary figures and images for: Spatiotemporal evolution of dissolved organic matter (DOM) and its response to environmental factors and human activities
Source: PLoS One. 2023 Oct 11;18(10):e0292705. doi: 10.1371/journal.pone.0292705 (PMC10566700; doi:10.1371/journal.pone.0292705)

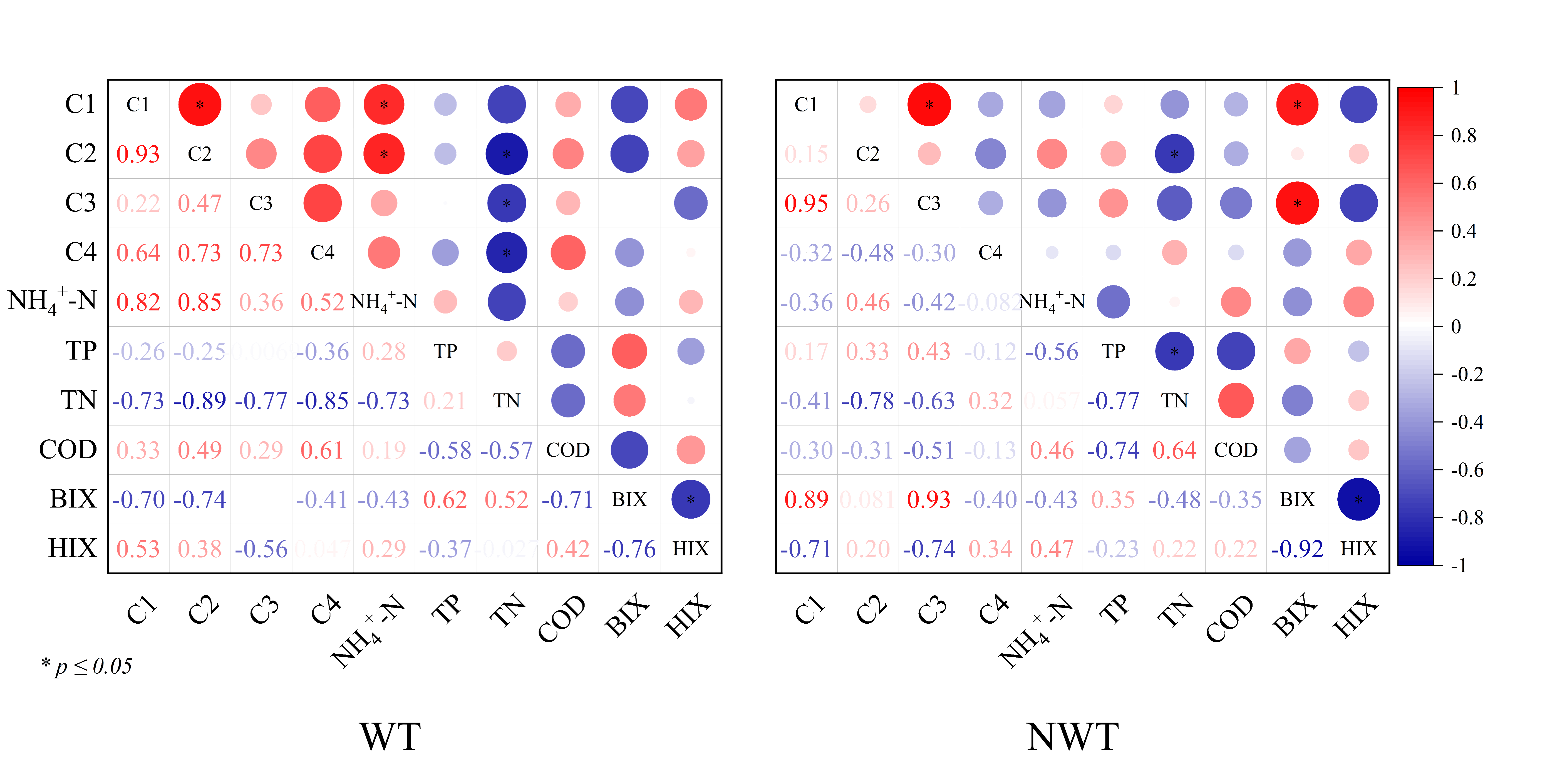

Supplement: S2 Fig — (TIF) [file pone.0292705.s002.tif]

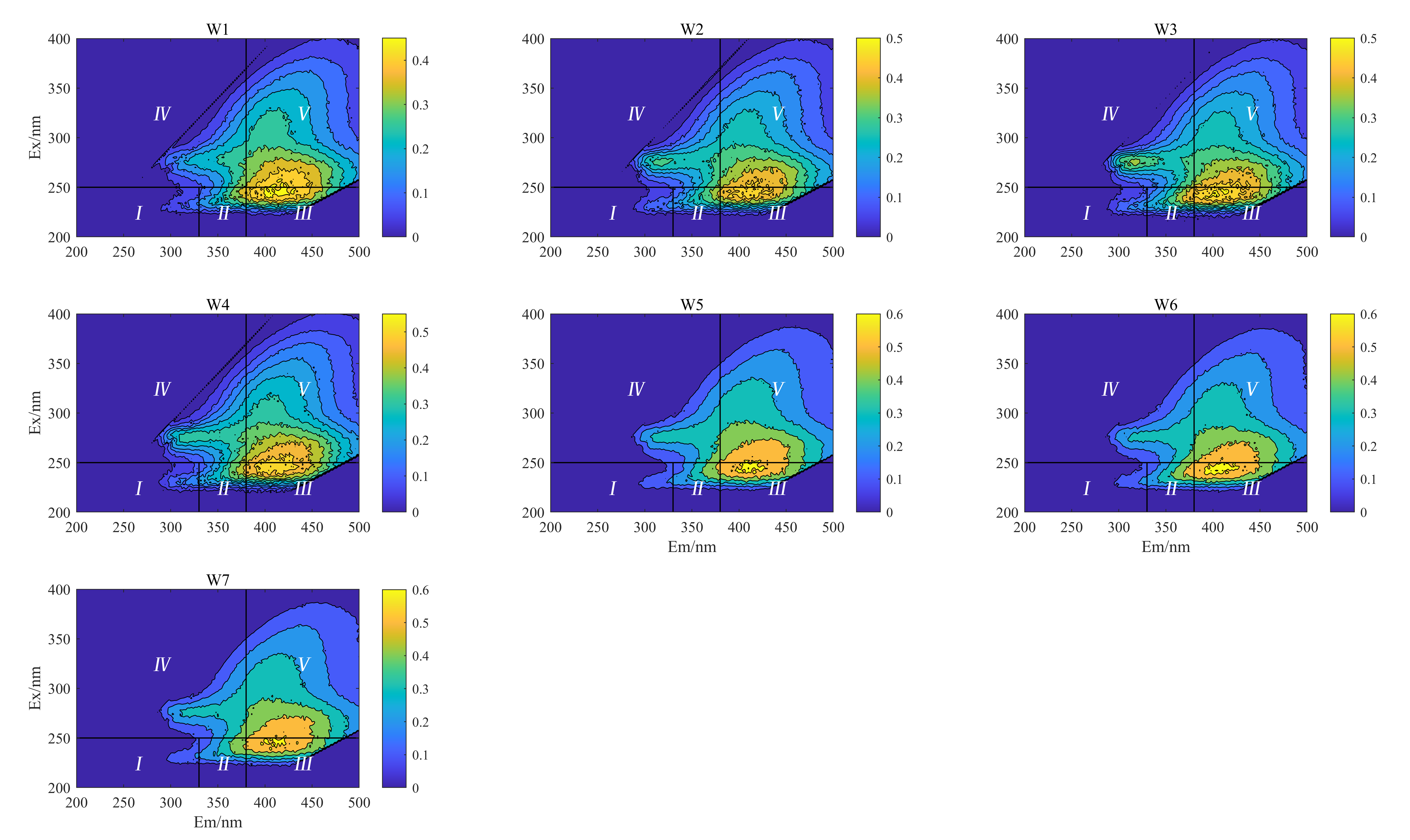

Supplement: S4 Fig — (TIF) [file pone.0292705.s004.tif]

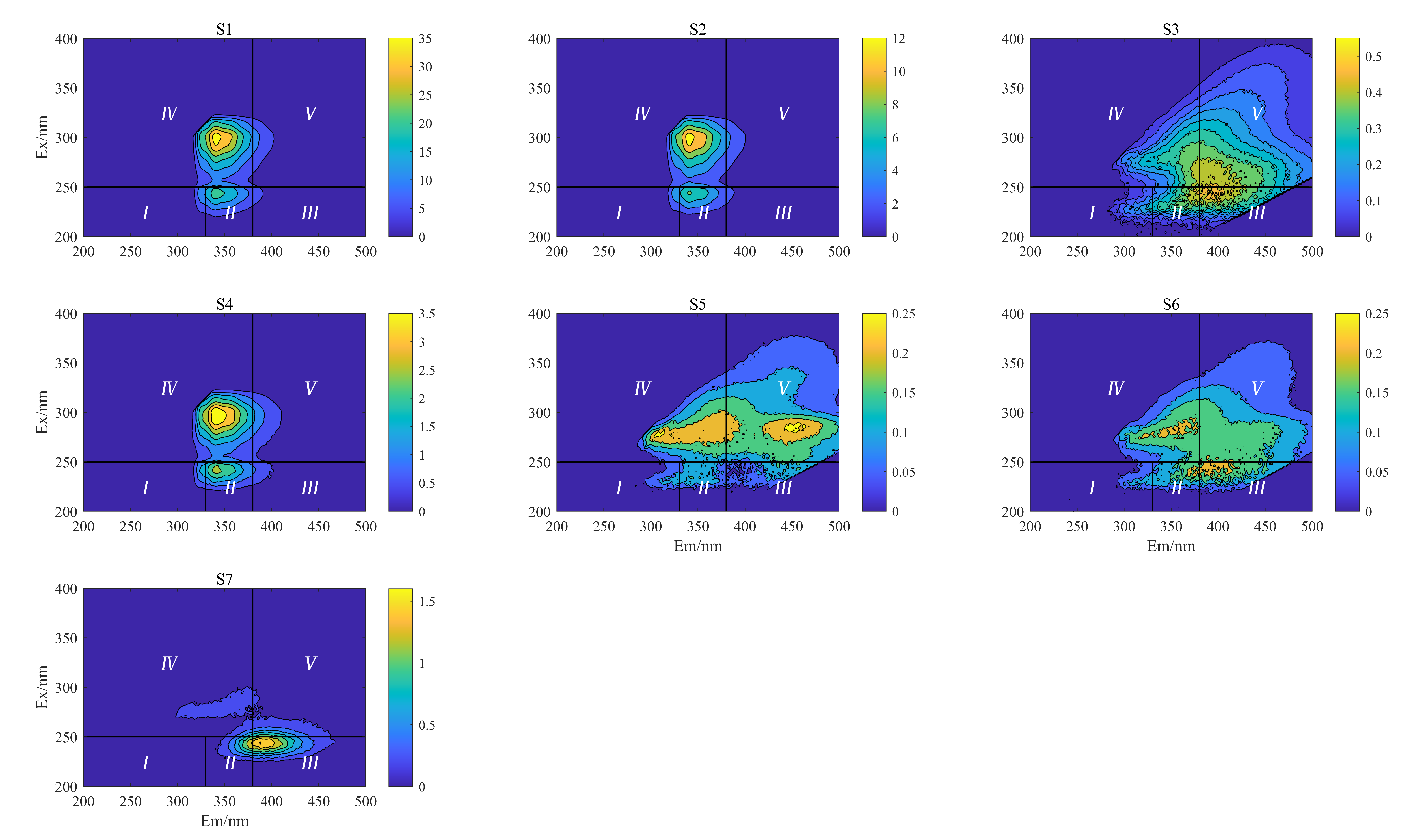

Supplement: S5 Fig — (TIF) [file pone.0292705.s005.tif]
