# Supplementary material for: Spatiotemporal evolution of dissolved organic matter (DOM) and its response to environmental factors and human activities
Source: PLoS One. 2023 Oct 11;18(10):e0292705. doi: 10.1371/journal.pone.0292705 (PMC10566700; doi:10.1371/journal.pone.0292705)
Supplement: S1 Table — (DOCX) [file pone.0292705.s006.docx]

**Table S1** Water quality parameters and reference methods used

| Parameter (Abbreviation) | Reference Method | |
| --- | --- | --- |
|  | Methodology | Method Numbers |
| Chemical oxygen demand (COD) | Dichromate method | HJ 828—2017 |
| Ammonia nitrogen (NH_4_^+^-N) | Nessler’s reagent spectrophotometry | HJ 535-2009 |
| Total nitrogen (TN) | Alkaline potassium persulfate digestion UV Spectrophotometric method | HJ 636—2012 |
| Total phosphorus (TP) | Ammonium molybdate spectrophotometric method | GB 11893-1989 |
